# Topical Anaesthesia Using a Soft Mist Spray Device Allows Comfortable Awake Visualisation of the Airway via Self-Videolaryngoscopy in Volunteers

**DOI:** 10.3390/medicina60010176

**Published:** 2024-01-19

**Authors:** Hielke Markerink, Geert-Jan van Geffen, Jörgen Bruhn

**Affiliations:** Department of Anesthesiology, Pain and Palliative Medicine, Radboud University Medical Centre (RUMC), 6525 GA Nijmegen, The Netherlands

**Keywords:** medical device, topical airway anaesthesia, videolaryngoscopy

## Abstract

*Background*: During endotracheal intubation, there is a 10% incidence of difficult laryngoscopy, which may result in serious complications. It is important to obtain as much information about the visibility of laryngeal structures before the patient is anaesthetised. Performing awake (video-) laryngoscopy on a patient is uncomfortable and can trigger gagging and coughing reflexes, making visualisation nearly impossible. The objective of this study is to evaluate the effectiveness of a soft mist spray device for airway anaesthesia during awake (video-) laryngoscopy. *Methods*: Twenty healthy volunteers inhaled through the Trachospray device, which was placed in their mouths. Two 2 mL syringes containing lidocaine at 4% were sprayed into the airway during inspiration. After several minutes, the subjects were asked to perform a videolaryngoscopy on themselves until the glottic structures and the vocal cords were visible. Upon completion of the procedure, all participants were asked to fill out a feedback form. *Results*: The duration of the videolaryngoscopy to visualisation of the vocal cords averaged 17 ± 13 s. After analysing the data, three distinct groups emerged as follows: Group 1 (70% of participants) showed no response, allowing for easy insertion of the videolaryngoscope. Group 2 (25% of participants) exhibited a light response but still permitted easy insertion and visualisation. One patient demonstrated a clear response with noticeable laryngeal contraction, requiring slightly more effort and discomfort for insertion. In 80% of the participants, the laryngeal structures were visualised according to Cormack–Lehane grade 1. All participants reported a high level of comfort, with an average rating of NRS 8. The anaesthesiologist assessed the level of anaesthesia as good to very good. No adverse events were observed. *Conclusions*: The Trachospray provided good, reliable, comfortable, and safe topical anaesthesia for awake videolaryngoscopy. This enables a direct visual assessment of the airway and may assist in making decisions regarding airway management for tracheal intubation.

## 1. Introduction

Videolaryngoscopy is often used for endotracheal intubation, which is a medical procedure that involves inserting a tube through the mouth or nose and into the trachea to establish a secure airway. Proficiency in this technique requires training, experience, and regular updates to maintain competence. Unfortunately, a physical examination is not a reliable indicator of whether intubation will be easy or difficult. Difficulty in laryngoscopy occurs in about 10% of cases and can lead to serious complications [1,2]. Intubation under general anaesthesia always carries the risk of losing control of the airway, posing a significant danger to the patient. Therefore, it is clinically important to have a fast and easy way to assess the visibility of laryngeal structures and vocal cords during (video-) laryngoscopy before the patient is anaesthetised. This can help to identify patients with a difficult airway and reduce the risk of complications during intubation.

Performing laryngoscopy on an awake patient without complete anaesthesia of the airway can be uncomfortable and lead to excessive salivation, gagging, and coughing reflexes. These reflexes can make intubation nearly impossible. The anaesthesia of the airway can suppress these reflexes and facilitate the procedure, particularly due to the maintenance of muscular tone.

Previous studies have demonstrated that the Trachospray soft mist spray device ensures even coverage of local anaesthetics in the mouth, hypopharynx, and vocal cords, and hence, more effective topical anaesthesia [3]. This adequate level of anaesthesia of the upper airway enables a comfortable awake passage through the vocal cords via a flexible bronchoscopy [4].

This interventional study is designed to evaluate the clinical performance of the soft mist spray device (Trachospray) for airway anaesthesia during awake (conscious) videolaryngoscopy.

## 2. Methods

This interventional study was reviewed and approved by the Medical Research Ethical Commission of the Radboud University Medical Centre (Radboudumc), Nijmegen, The Netherlands. This study was registered under number NL 78481.091.21 and was conducted between August 2022 and February 2023. It was registered on ClinicalTrials.gov with the ID: NCT05478122. All healthy volunteers provided written informed consent before participating. A total of twenty healthy volunteers were recruited for the study. Volunteers between the ages of 18 and 60 years, with a lean body weight of ≥50 kg and ASA physical status 1, were eligible for participation. Exclusion criteria included a history of hepatic, renal, and coagulation disorders, respiratory tract pathology, pregnancy or lactation, risk of regurgitation or aspiration, an allergy to amide-type local anaesthetics, an inability to cooperate with an adequate airway assessment, and lack of written informed consent. Due to the pilot construction of this interventional study, no sample size calculation was performed.

Before the application of topical anaesthesia, each subject was instructed using a manikin on how to use a GlideScope videolaryngoscope (GlideScope, Verathon, Bothell, WA, USA), the required manoeuvres and the corresponding views on the monitor of the videolaryngoscope. During the self-videolaryngoscopy, a trained anaesthesiologist stood next to the volunteer, ready to coach and advise the volunteer if needed to improve the procedure.

**Figure 1:**
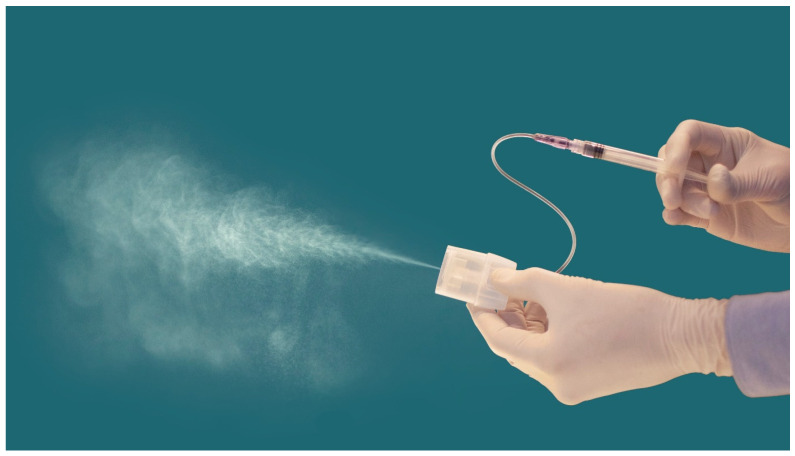
Trachospray soft mist demonstration.

Two 2 mL syringes containing lidocaine at 4% were prepared and connected to the Trachospray device (Figure 1). The inhalator was placed in the subject’s mouth. To achieve an optimal distribution pattern, the subject was asked to inhale 2× 1 mL normally, 2× 0.5 mL deeply, and 2× 0.5 mL shortly until the total amount of local anaesthetic had been administered.

**Figure 2:**
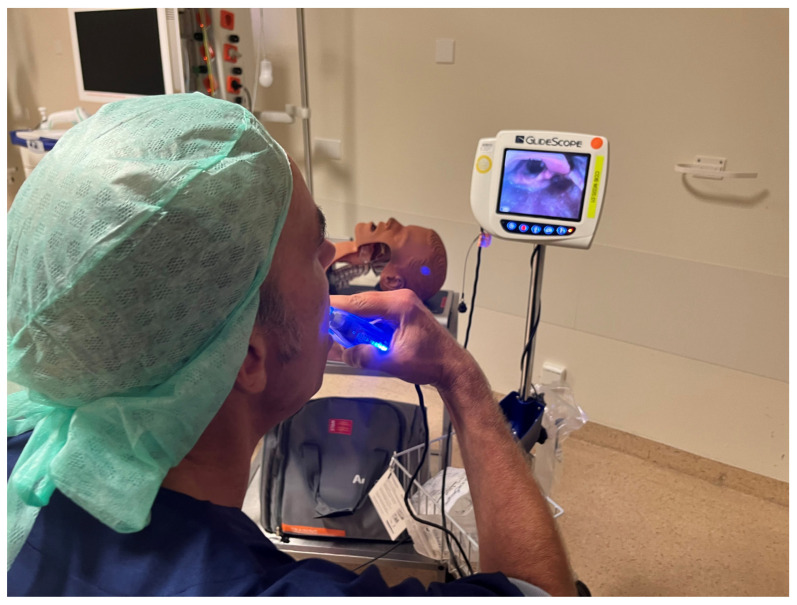
Research set-up.

During inspiration, lidocaine was sprayed by the attending skilled anaesthesiologist while the subject was asked to exhale through the nose. After several minutes, the subject was asked to perform the videolaryngoscopy by themselves (to keep them in complete control) until the glottic structures and the vocal cords were visible on the monitor (Figure 2). An observer not involved in the procedures classified the view according to the Cormack–Lehane classification system [5] and documented the subject’s possible reactions (e.g., coughing, gag reflex).

Before leaving the hospital after the procedure, all participants were asked to complete a feedback form. They were asked to rate the method of anaesthesia and the comfort level on a scale of 1 to 10. The attending anaesthesiologist was asked to rate the level of anaesthesia. All data and recordings were stored in an electronic data capture platform while maintaining anonymity. Prior to analysing the data, an independent qualified monitor conducted three monitoring visits to ensure data accuracy. Statistical analysis was performed using SPSS 27 software for Windows (SPSS Inc., Chicago, IL, USA). Descriptive statistical methods were used (such as the frequency count, mean, and standard deviation).

## 3. Results

Twenty healthy volunteers were enrolled in this interventional study, all of whom met the inclusion and exclusion criteria. The demographic information of these participants is presented in Table 1.

Various time measurements were recorded during the entire procedure. The average total spraying time was 171 ± 17 s (mean ± SD), while the duration from the start of videolaryngoscopy to visualising the vocal cords averaged 17 ± 13 s (mean ± SD).

Upon reviewing the data, the results could be categorised into three groups. Group 1 consisted of individuals (n = 14, 70% of the total) who exhibited no response, allowing for easy insertion of the videolaryngoscope into the pharynx. Group 2 included participants (n = 5, 25% of the total) who showed a light response characterised by mild laryngeal contraction, still enabling easy insertion of the videolaryngoscope. Lastly, Group 3 consisted of one participant (n = 1, 5% of the total) who demonstrated a clear response with noticeable laryngeal contraction, requiring some effort and causing discomfort during the insertion of the videolaryngoscope.

The view of the glottic structures and vocal cords was classified according to the Cormack–Lehane classification system as grade 1 (n = 16, 80% of the total), grade 2a (n = 3, 15% of the total), or grade 2b (n = 1, 5% of the total).

Upon the completion of the procedure, all participants were asked to complete a feedback form. The volunteers reported the method of anaesthesia as comfortable to very comfortable. The level of comfort was high, with an NRS rating of 8 (0 = no comfort, 10 = very comfortable). The anaesthesiologist reported the level of anaesthesia of the airway generally as good to very good (see Table 2).

No adverse or serious adverse events occurred during this study.

## 4. Discussion

This interventional study aimed to evaluate the clinical performance of a soft mist spray device, the Trachospray, for airway anaesthesia during awake (conscious) videolaryngoscopy. The Trachospray device provided good, reliable, and safe anaesthesia for awake videolaryngoscopy with high comfort for the subject. It enabled fast and comfortable awake videolaryngoscopy.

A systematic review involving 33.559 patients revealed the overall incidence of difficult intubation to be 10% [1]. Administering general anaesthesia with endotracheal intubation (ETI) to a patient with a known difficult airway often leads to stress for the anaesthesiologist while posing potential dangers to the patients. The Difficult Airway Society (DAS) recommends awake tracheal intubation when predictors of difficult airway management are present [6].

For these patients, it is clinically relevant to have a rapid and straightforward method to gain more information regarding the visibility of laryngeal structures and the vocal cords during (video) laryngoscopy before administering anaesthesia. Ideally, (video) laryngoscopy should be performed prior to general anaesthesia. The magnified views on the videolaryngoscopy screen allow improved spatial awareness and a larger field of view, aiding the recognition of airway anatomy and the direct observation of airway manipulations (such as the suction and application of additional topical anaesthesia) [7].

The main problem for awake (video) laryngoscopy is the gag reflex. The gag reflex is a normal defense mechanism for survival by preventing possibly harmful foreign bodies from entering the trachea, pharynx, or larynx. The gag reflex is influenced by five types of stimuli. These include olfactory or taste stimuli, which can be linked to the smell or taste of materials used in treatment or during procedures. Acoustic stimuli are triggered by the noise of instruments or other procedure-related noise, while mechanical stimuli result from direct instrumentation in the mouth or hypopharynx, targeting specific trigger zones. Visual stimuli can be induced by the mere sight of instruments or materials, and psychic stimuli arise from the patient’s fear and anxiety, often stemming from previous unpleasant experiences or psychological factors [8]. We hypothesised that allowing subjects to independently handle and control the videolaryngoscope might mitigate the impact of visual and psychic stimuli that typically provoke a gag reflex. This attention–diversion method approach could provide a more reliable assessment of the effectiveness of topical anaesthesia in blocking the gag reflex induced by mechanical stimuli.

Several videos on YouTube exist where individuals perform a videolaryngoscopy on themselves. This might be an innovative new clinical approach. But this is not (yet) clinical practice, and the clinical applicability is not defined yet. It is important to note that results may be different when a laryngoscopy is performed by another person.

After a direct visual assessment of the airway, a more deliberate decision can be made regarding the technique of visualisation and the depth of sedation during the airway manipulations for tracheal intubation.

Interestingly, this important information is not easily available otherwise. Several clinical predictors for difficult intubation have been described, like the Mallampatti score, mouth opening, thyromental distance, and sternomental distance. It might be clinically quite easy to judge a very easy or a very challenging airway. But there is a large grey area where the clinical predictors for a difficult airway only have relatively low sensitivity and specificity [9]. When a CT of the neck region is available, a computerised reconstruction of a virtual bronchoscopy/laryngoscopy might be possible [10,11,12]. This might give valuable information about a fibreoptic approach to the airway but much less about the laryngeal view with a classical or video laryngoscopy. Thin, flexible rhinolaryngoscopes are often used by ENT doctors (and less often by anaesthesiologists [13]) to obtain information about oropharyngeal tumours, swelling, or the (distorted) anatomy of the laryngeal area. Nevertheless, this only provides very limited information about the laryngeal view with a classical or video laryngoscopy. Classical direct laryngoscopy with topical anaesthesia and sedation only is of very limited value in clinical anaesthesia. The relatively flat shape of the Macintosh laryngoscope exerts a high pressure on the (base of the) tongue which, directly activates the gagging reflex even in the presence of (superficial) topical anaesthesia. The more curved blade design of, e.g., the Glidescope videolaryngoscope, in combination with the camera chip in the tip, significantly reduces the need for exerted pressure on the tongue to obtain a good laryngeal view. The angulation of the blade of the videolaryngoscope seems to be important for smooth awake videolaryngoscopy. With our topical anaesthesia set-up, as described in this study, we obtained a fast and good view of the larynx. But the angulated blade might lead to the anterior position of the larynx [14]. Then, for the easier introduction of the tracheal tube, the use of a stylet [15] or, e.g., a Frova intubating introducer, is advantageous.

In the future, the recently introduced VideoStylet [16] might present an interesting option for this approach. With its flexible tip, it might be easier to navigate than, e.g., the Bonfils scope. Its shape enables navigation to the larynx without putting pressure on the tongue. When the larynx is ‘a vue’ with the VideoStylet, the pre-loaded tracheal tube can be easily introduced into the trachea.

It is important to note that our pilot study did not include patients with respiratory tract pathology. Future studies are warranted to investigate whether our results are also valid for patients with respiratory tract pathology and patients with an anatomically difficult airway.

In our study, participants performed videolaryngoscopy after topical anaesthesia of the airway without additional medication. Previous in vitro investigations using an airway model, specifically the Alberta Idealised Throat connected to a Next-Generation Impactor, demonstrated that the Trachospray soft mist spray device achieved the uniform distribution of local anaesthetics in the mouth, hypopharynx, and vocal cords region [3]. In contrast, currently available topical anaesthesia applicators generate droplets that are either too small, too big or provide inadequate flow patterns [3]. These small droplets result in thin airway coverage but high lung deposition, while larger droplets primarily settle in the hypopharynx. As a result, accurate delivery of the local anaesthetic and reaching the intended target areas appears to be considerably suboptimal with existing devices.

The optimal concentration and volume of local anaesthetic required for optimal use with this device and different applications have not been established. However, it was found that a dosage of 160 mg of lidocaine provided sufficient topical anaesthesia in most participants, which is only half the maximum recommended concentration for lidocaine topical anaesthesia (4–5 mg/kg) in a person weighing 70 kg. Studies have indicated that doses of topical lidocaine up to 9 mg/kg are safe for fibreoptic bronchoscopy [17]. Therefore, the amount and concentration of lidocaine employed in this study can serve as a starting point for further clinical evaluations. Nonetheless, it would be intriguing to explore whether altering the concentration of lidocaine in either direction would impact the outcomes. The Difficult Airway Society (DAS) guidelines for awake tracheal intubation in adults suggest that lower concentrations of lidocaine might be as effective as higher concentrations, but higher concentrations may be associated with a more rapid onset of airway anaesthesia [6].

Exploring the impact of different breathing patterns on the distribution of local anaesthetics presents an intriguing avenue for investigation. In this study, participants were instructed to inhale 2× 1 mL normally, 2× 0.5 mL deeply, and 2× 0.5 mL shortly. We hypothesise that during deep inhalations, the local anaesthetic is likely to be deposited on the posterior wall of the pharynx due to the high velocity of the moving droplets. Conversely, with short inhalations, the droplets are more likely to settle in the mouth region. However, further research is warranted to fully understand the effect of inhalation patterns on the deposition of a local anaesthetic in the upper airway.

## 5. Conclusions

In conclusion, the findings of this intervention study indicate that the soft mist spray device effectively and rapidly achieves a satisfactory level of airway anaesthesia. This facilitates comfortable awake (conscious) videolaryngoscopy and allows a direct visual assessment of the airway, and may guide decisions regarding airway management for tracheal intubation.

Fibreoptic intubation has previously been shown to be possible with topical airway anaesthesia using the Trachospray device. But videolaryngoscopy is faster and easier to learn and use. The area of contact and the pressure on airway structures is different for videolaryngoscopy compared to a fibreoptic approach. Our findings showed that this might also be adequately covered by topical airway anaesthesia with the Trachospray device.

## Figures and Tables

**Table 1:** Demographic characteristics of the study population.

Characteristic	
Male (n)	10 (50%)
Female (n)	10 (50%)
Age (y, mean)	27.05 (SD 5.23)
Weight (kg, mean)	73.10 (SD 13.15)
Length (cm, mean)	177.50 (SD 10.27)
BMI (kg/m^2^, mean)	23.07 (SD 2.72)

**Table 2:** Reports by volunteers and anaesthesiologist.

Characteristic	Result
Comfort level (NRS, mean)	8 (SD 0.6)
Level of anaesthesia (NRS, mean)	9 (SD 1)

## Data Availability

Dataset available on request from the authors.
